# Publish With Undergraduates or Perish?: Strategies for Preserving Faculty Time in Undergraduate Research Supervision at Large Universities and Liberal Arts Colleges

**DOI:** 10.3389/fpsyg.2019.00828

**Published:** 2019-04-17

**Authors:** Jeanine K. Stefanucci

**Affiliations:** Department of Psychology, University of Utah, Salt Lake City, UT, United States

**Keywords:** undergraduate publication, research mentoring, faculty time allocation, writing training, mentoring undergraduates

To promote “unique” undergraduate experiences, both research-intensive universities (designated R1 by the Carnegie Classification of Institutions of Higher Education) and liberal arts institutions (called Baccalaureate Colleges by the Carnegie Classification) are keen to involve undergraduates in faculty research projects (Masterson, 2017). Students who engage in these activities certainly receive a more enriching education than those who do not (Lopatto, 2007; Russell et al., 2007). Faculty may also experience a boost in morale from engaging in research with undergraduates (Wayment and Dickson, 2008). But how much time do undergraduate publications take for all involved? How do faculty at research-focused institutions determine whether they should publish with undergraduates? Here I present two strategies for ensuring efficiency in accomplishing publications with undergraduates. I believe that these strategies apply to faculty at R1 institutions as well as those at liberal arts/baccalaureate colleges with fairly high research expectations. I have been a faculty member at both types of institutions, including the University of Utah and the College of William and Mary. Obviously certain strategies will not work for every type of campus, but I do feel that the strategies I propose are effective in the different research environments present at R1 and liberal arts institutions with high research expectations. I argue that research collaboration with undergraduates if done wisely, helps advance one's own research agenda (see also Petrella and Jung, 2008), thereby making preliminary work for grants and also publications with undergraduates helpful not only for possibly obtaining grants but also in achieving promotion or tenure. Further, these strategies can ensure productivity and protect faculty time while rewarding students and faculty alike.

My strategies aim to make the process of publishing with undergraduates as efficient as possible. Many factors play a role in the time taken to get from start to finish on an undergraduate publication. For example, the time needed for undergraduates and faculty mentors to develop a functional and productive team varies. Other factors include the level of preparation and motivation in the undergraduates themselves, the support structures (or lack thereof) for undergraduate research, and the faculty mentor's obligations. I am not the first to bring up the time obstacles that can plague success in undergraduates publishing their work (Wendt, 2006). However, I feel that more discussion should ensue across institutions and experiences in order to support faculty (especially those at the junior level) who decide to mentor undergraduates in publishing. After all, mentoring undergraduates in publishing can be extremely rewarding!

## Strategy 1: Delegate or Drown

Professors at all types of institutions are increasingly asked to do more for the same salary and incentives (Milem et al., 2000; Link et al., 2008). Tenure-track faculty at liberal arts institutions are no longer expected simply to be excellent teachers; they are now expected to write grants to obtain extramural funding (Webb, 2008). Grant applications obviously take time, as does preliminary research to support proposals and a research group that can actually complete the work if funding is awarded. What should a faculty member consider before mentoring an undergraduate? The obvious first consideration should be the overall time commitment required given the professor's schedule, although the student's available time is relevant, too. Both the undergraduates and faculty need to be committed to the research in order for publications to result, but faculty may need to delegate some responsibilities to ensure efficiency.

The overall strategy of delegating involves several specific tactics. First, assume at least an hour of time per week to meet with each undergraduate. In my experience, regular check-ins with the undergraduates are necessary to gauge their level of expertise and to keep them focused and on track to complete research in a timeframe that will help rather than burden faculty. This meeting might be part of a weekly lab meeting where all students convene to “report” on their projects, but having more time available is useful for students who need specific attention or more feedback. However, it is not a good idea to suggest there is unlimited time for undergraduates!

Toward this end, I have found that at both types of institutions (liberal arts and R1), it is helpful to show students what they will be undertaking in terms of commitment and time. To do so, I ask prospective students to join the weekly meetings in order to see other students' progress. If all goes well, prospective students then decide to aid an already existing project (and earn themselves second or third authorship instead of first). Once they know other students in the lab, they may decide to assist ongoing projects, which can result in more senior students mentoring junior students (even at the undergraduate level). This situation is especially useful if one does not have graduate students available for mentoring. I often tell the “new” undergraduate students that we will evaluate their performance over the course of the semester in order to determine if we are willing to allow them to continue toward a potential publication. I also ask them to consider whether they have the time and energy to commit to conducting research for publication.

Finally, it is also wise to ask students to write small sections of papers or project reports to gauge their writing skills, as these are excellent predictors of whether they can carry a project through to publication. At larger institutions, graduate students are able to take on some of this early mentoring and can, in the process, help faculty identify which students are better suited for research and publication. The graduate students often ask the undergraduates to take a first pass at writing a method section and, in turn, offer them later authorship on a paper if we use this writing after revisions. Graduate students can also spearhead conducting preliminary data analyses with students if the undergraduates are less familiar with data processing and software.

One difference that I have experienced between large research institutions and smaller liberal arts colleges is that capstone courses requiring independent research projects for the major are more prevalent at the latter. Such courses allow faculty to “double dip” on teaching and research in that they can conduct research in classes with undergraduates that may lead to publications. The College of William and Mary had such a course; undergraduates designed and conducted experiments over the semester and then wrote up their findings in manuscript form. Two of these projects resulted in publications for my laboratory (see Geuss et al., 2010; Stefanucci et al., 2012). These courses allow for delegation of research activities given students may use class time to write sections of papers or meet to talk about research. In addition to capstone courses, options allowing students to conduct independent research projects with faculty for course credit can also include mentoring Honors students in writing and conducting theses. Honors students are self-motivated to complete a publication: their thesis. They also often have better training in writing from Honors-specific courses that usually require higher amounts of writing than their non-Honors counterpart.

So, to summarize this section, if you plan to publish with undergraduates, delegate as much of the mentoring as you can. In addition, reach out to students completing a capstone project (even if not yours) or writing an honors thesis. Whenever possible, try to double up on required tenure activities (i.e., teaching and research) to save time!

## Strategy 2: Get Credit When Credit Is Due

If you are going to invest time in mentoring undergraduates toward publication, then you need to make sure that your institution gives you the appropriate credit and recognition. Yes, publishing with an undergraduate is highly rewarding in and of itself (which is why we do it!), but it is nonetheless important to convey your level of commitment to your colleagues, especially if you are junior. This is even more important if undergraduate publications are a new “requirement” at your institution. The following section discusses ways in which various types of institutions credit undergraduate publication and offers suggestions for how to ask for credit if it is lacking.

In the interest of productivity and time, aim to make research supervision that results in undergraduate publications count as part of your teaching load. It is wise to keep track of the time you spend supervising outside research so that you can demonstrate whether it approaches (or exceeds!) the time you spend teaching a class. Some larger research institutions now equate mentoring to a class if the hours are commensurate; if your institution is not one of them, then this issue may be worth bringing up with fellow faculty or administration.

Another issue to raise with colleagues is how your institution weighs undergraduate publications for promotion and tenure. At the College of William and Mary, such publications were expected and given much weight at faculty reviews. My current R1 institution, the University of Utah, does not weight papers published with undergraduates any differently than those published with graduate students. So, if you are at an institution that deemphasizes undergraduate research activities, aim to encourage committees to change this practice. Doing so will make investing in publications with undergraduates a better use of time.

In addition to the credit that you are due for mentoring, also consider the credit that your undergraduate students should be given for their work (such as authorship order for publications and presentations). The appropriate level of credit here can sometimes be hard to discern (especially when graduate students may be interacting with the students more regularly than you). At the very least, however, you and your students should be clear from the outset what you both hope and expect to get out of publishing—and how you will divide the labor and the author credits. This matter of authorship is especially important to discuss if you are junior faculty who need to have more first author publications before going up for tenure. I often candidly discussed the issue of authorship with my students when I was junior faculty, and I still do so today. We would agree upon the amount of work that would warrant first authorship as well as who had the original idea for the work. Then we would decide on a preliminary authorship order. Sometimes authorship can and should change in the process of writing, but we agreed that all parties would meet in person if someone felt authorship should be changed from the original arrangement. Again, this plan allows for flexibility if students end up doing more or less than planned. My current graduate students are also expected to mentor undergraduates in research for publication whenever possible. Doing so allows them to show evidence of effective and productive mentorship for post-doctoral or job applications, which as I have said already is becoming more important for academic positions.

To conclude, the second primary strategy—getting credit where credit is due—helps make sure that your institution and colleagues do not overlook time invested in undergraduate publications. Instead, ideally, this time and investment should garner you more credit during faculty reviews and should garner your students the proper credit—as well as an excellent intellectual experience.

## Conclusions

I believe that publishing with undergraduates is one of the most rewarding aspects of faculty life. However, faculty need to consider how to preserve time when undertaking undergraduate publication. Overall, I suggest finding ways to delegate mentoring and also making sure that your efforts are rewarded. Doing so will help you publish with undergraduates without perishing from lack of time.

## Author Contributions

The author confirms being the sole contributor of this work and has approved it for publication.

### Conflict of Interest Statement

The author declares that the research was conducted in the absence of any commercial or financial relationships that could be construed as a potential conflict of interest.

## References

[B1] Geuss M. Stefanucci J.K. de Benedictis-Kessner J. Stevens N. R. (2010). A balancing act: physical balance, through arousal, influences size perception. Attent. Percept. Psychophys. 72, 1890–1902. 10.3758/APP.72.7.1890 20952786 PMC3298363

[B2] Link A. N. Swann C. A. Bozeman B. (2008). A time allocation study of university faculty. Econ. Edu. Rev. 27, 363–374. 10.1016/j.econedurev.2007.04.002

[B3] Lopatto D. (2007). Undergraduate research experiences support science career decisions and active learning. CBE Life Sci. Edu. 6, 297–306. 10.1187/cbe.07-06-0039 18056301 PMC2104507

[B4] Masterson K. (2017). Expanding Undergraduate Research. Available online at: https://www.chronicle.com/ (accessed October 8, 2017).

[B5] Milem J. Berger J. Dey E. (2000). Faculty time allocation: a study of change over twenty years. J. Higher Edu. 71, 454–475. 10.2307/2649148

[B6] Petrella J. K. Jung A. P. (2008). Undergraduate research: importance, benefits, and challenges. Int. J. Exer. Sci. 1, 91–95. 27182299 10.70252/MXRI7483 PMC4739295

[B7] Russell S. H. Hancock M. P. McCullough J. (2007). Benefits of undergraduate research experiences. Science 316, 548–549. 10.1126/science.1140384 17463273

[B8] Stefanucci J. K. Gagnon K. T. Tompkins C. Bullock K. E. (2012). Plunging into the pool of death: imagining a dangerous outcome influences distance perception. Perception 41, 1–11. 10.1068/p7131 22611659 PMC3359865

[B9] Wayment H. A. Dickson K. L. (2008). Increasing student participation in undergraduate research benefits students, faculty, and department. Teach. Psychol. 35, 194–197. 10.1177/009862830803500307

[B10] Webb S. A. (2008). Liberal arts college faculty: finding the sweet spot. Science 10.1126/science.caredit.a0800096

[B11] Wendt D. (2006). Publishing in psychology: an overview for undergraduate students. Intuition BYU Undergraduate J. Psychol. 2, 9–13.

